# New Insights into the Effects of Microbial Muramidase Addition in the Diets of Broiler Chickens

**DOI:** 10.3390/ani13081356

**Published:** 2023-04-15

**Authors:** Shimaa A. Amer, Mahmoud Farahat, Ahmed Gouda, Ahmed A. A. Abdel-Wareth, Abdel-Wahab A. Abdel-Warith, Elsayed M. Younis, Gehad Elsaid Elshopakey, Weam Mohamed Baher, Gehan K. Saleh, Simon J. Davies, Ghadeer A. Attia

**Affiliations:** 1 Department of Nutrition & Clinical Nutrition, Faculty of Veterinary Medicine, Zagazig University, Zagazig 44511, Egypt; 2 Animal Production Department, Agricultural & Biological Research Division, National Research Center, Dokki, Cairo 11865, Egypt; 3 Department of Animal and Poultry Production, Faculty of Agriculture, South Valley University, Qena 83523, Egypt; 4 Department of Zoology, College of Science, King Saud University, P.O. Box 2455, Riyadh 11451, Saudi Arabia; 5 Department of Clinical Pathology, Faculty of Veterinary Medicine, Mansoura University, Mansoura 53511, Egypt; 6 Food Hygiene Department, Animal Health Research Institute (AHRI) (Mansoura Branch) Agriculture Research Center (ARC), P.O. Box 246, Dokki, Giza 12618, Egypt; 7 Biochemistry Department, Animal Health Research Institute (AHRI) (Mansoura Branch) Agriculture Research Center (ARC), P.O. Box 246, Dokki, Giza 12618, Egypt; 8 School of Science and Engineering, National University of Ireland Galway Republic of Ireland, H91 TK33 Galway, Ireland

**Keywords:** blood biochemistry, broilers, growth performance, microbial muramidase, sensory characteristics

## Abstract

**Simple Summary:**

Adding exogenous enzymes in animal diets is a widespread technique with the advantages of lowering antinutritional compounds and increasing nutrient availability and performance. This study evaluated dietary microbial muramidase with four levels (0, 200, 400, and 600 mg Kg^−1^ diet, with enzyme activity 0, 12,000, 24,000, and 36,000 LSU(F)/kg diet, respectively) in broiler chicken production. The present study results concluded that MUR had no positive effect on the growth performance of the birds. Still, it can improve breast muscles’ fatty acid profile, immune status, and blood biochemistry of broiler chickens.

**Abstract:**

The study aimed to explore how broiler chickens’ blood biochemistry, breast muscles’ fatty acid profile, growth, intestinal morphology, and immune status would be influenced by adding microbial muramidase (MUR) to the diet. Four hundred 3-day-old male broiler chickens were allocated to a completely randomized design consisting of four nutritional treatments (*n* = 100 per treatment, 10 chicks/replicate), each containing MUR at levels of 0 (control group), 200, 400, and 600 mg Kg^−1^ diet, with enzyme activity 0, 12,000, 24,000, and 36,000 LSU(F)/kg diet, respectively. The 35-day experiment was completed. The findings showed that adding MUR to broiler meals in amounts of 200, 400, or 600 mg/kg had no impact on growth performance (*p* > 0.05) during the periods of 4–10, 11–23, and 24–35 days of age. MUR supplementation quadratically impacted the feed conversion ratio of broiler chicks at 11 and 23 days of age (*p* = 0.02). MUR addition to the diet significantly and level-dependently enhanced the percentage of n−3 and n−6 polyunsaturated fatty acids (PUFA) in breast muscles (*p* ≤ 0.01), with no alterations to the sensory characteristics of the breast muscles. Dietary MUR increased most of the morphometric dimensions of the small intestine, with the best results recorded at the 200 and 400 mg Kg^−1^ levels. MUR supplementation at 200, 400, and 600 mg kg^−1^ linearly lowered the total cholesterol, triglycerides, and low-density lipoprotein cholesterol level (*p* < 0.01). Still, it significantly increased the high-density lipoprotein cholesterol and very-low-density lipoprotein cholesterol contents compared with the unsupplemented group. Compared to controls, there was a substantial rise in the blood concentration of total protein, albumin, globulin, IL10, complement 3, and lysozyme activity as MUR levels increased (*p* < 0.01). Moreover, MUR addition significantly increased the immunoexpression of lymphocyte subpopulation biomarkers. We could conclude that MUR can be added to broiler chicken diets up to 600 mg kg ^−1^ to improve broiler chickens’ fatty acid profile in breast muscles, immunity, and blood biochemistry. MUR addition had no positive influence on the bird’s growth.

## 1. Introduction

Poultry farmers have emphasized food safety and bird welfare during the past ten years. Consumer stress pushes for removing synthetic feed additives and reducing unnecessary breeding techniques to protect the environment. Recent poultry production’s two main objectives are high feed efficiency and rapid growth rate. For ideal bird performance, critical aspects influencing gastrointestinal health, such as the bird’s genetic potential, diet quality, environmental circumstances, and disease prevalence, must be considered [1]. Also, the efficient function of the gastrointestinal system is especially critical in influencing the animal’s performance. An optimal gastrointestinal function has recently been described as a stable state in which the microbiota and the host’s intestinal tract are in mutual balance, permitting the maintenance of the essential physiological functions leading to better health, performance, and welfare [2]. The structure and metabolic action of the gastrointestinal microbiota have the most important influence on host health because of their effect on intestinal physiology, nutrient absorption, immune response restitution, and, thus, resistance to pathogen colonization [3,4].

In animal nutrition, dietary supplementation with exogenous enzymes is a widespread technique. Enzymes are crucial in lowering antinutritional components and increasing the availability of the indigestible portions of the meal for absorption by the animal. Enzyme supplementation can maximize the nutritional content of meals, lowering feed costs, enhancing animal performance throughout growth, and lowering environmental pollution [5].

Peptidoglycans (PGNs) comprise the bacterial cell wall and are considered maintained products of the metabolism and activity of bacteria in the gastrointestinal tract. During normal bacterial cell wall recycling, enormous PGNs are shed from bacterial cell wall fragments in the gut so that PGNs can have a pro-inflammatory effect on the host’s gastrointestinal tract [6,7].

Muramidase (MUR; EC 3.2.1.17; N-acetylmuramidase or lysozyme) is a glycosyl hydrolytic enzyme that slashes the β-1, 4 glycosidic linkages between N-acetylmuramic acid and N-acetyl glucosamine in the carbohydrate backbone of bacterial PGNs. The primary dissociation products of PGNs are muropeptides that have been informed to alter the inflammatory response in the gastrointestinal tract of various species. But, the extent and form of inflammatory responses modified by mRNA peptides differ according to the cell type [8]. The best common muramidase is abundantly present in hen egg whites (HEW), but several MURs can be noticed in various kinds of secretions from plants, sperm, or microorganisms [9]. It has been shown that dietary muramidase has positive effects on chickens [10], rabbits [11], and pigs [12,13], which are largely due to the alteration in the gastrointestinal microbiota [14]. Muramidases also have immunomodulatory functions that have been established in livestock [15,16]. MUR hydrolyzes PGN-containing bacteria cell debris, which can improve digestive and absorptive functions and positive alteration of the intestinal inflammatory response, with subsequent enhancements in gut health and broiler performance [5,17]. Research on poultry and pig described the valuable effects of HEW lysozyme feeding on the gut microorganisms, involving a decline in pathogenic bacteria such as *Escherichia coli*, *Clostridium perfringens,* etc., on the antioxidant status of the gut, involving a rise in intestinal glutathione peroxidase gene expression, and on non-specific immunity in the gastrointestinal tract, involving a rise in gene expression of intestinal interleukin 10 and 18, and interferon-gamma [10,13,15]. Boroojeni et al. [18] postulated that adding microbial muramidase in broiler diets may enhance gastrointestinal function, resulting in improved nutrient digestion and absorption and better growth performance. Dietary MUR supplementation in broiler chicken diets improved the growth performance and increased the cecal number of *Lactobacillus* spp. [9]. Microbial enzymes display a variety of biological outcomes, such as immunomodulatory, antibacterial, and antioxidant properties [5,18,19].

More attention has been paid in recent years to the expansion of microbial enzymes as potential replacements for antibiotic growth promoters. Several studies have also been performed, with varying degrees of success, on how dietary enzymes impact poultry productivity [10,20,21,22,23,24]. However, several factors, such as composition, administrative levels, durations, and methodologies, as well as the age of the birds, affected how the poultry reacted to the enzymes [19,25]. The amount of study on the effects of microbial MUR on productivity and meat quality in broilers is still limited. Therefore, this study aimed to evaluate the possible impact of using MUR as a feed additive on the productive performance of broiler chicken by evaluating the bird’s growth, the effects on the meat quality measures such as fatty acid profile and sensory characteristics, intestinal histomorphology, serum lipid profile, immune status, and immunoexpression of lymphocyte subpopulation biomarkers.

## 2. Materials and Methods

### 2.1. Birds, Experimental Design, and Diets

This research was conducted in a poultry research unit at the faculty of veterinary medicine at Zagazig University, Zagazig, Egypt. Microbial muramidase, EC Number 3.2.1.17, lysozyme or N-acetylmuramidase, is produced by fermentation with a genetically modified strain of *Trichoderma reesei* (Accession number DSM 32338), Balancius™, Isando. The enzyme activity in the product is 60000 LSU(F)/g. Microbial muramidase’s (Balancius™) safety for chicken feeding was previously established [26].

Four hundred 1-day-old male Ross 308 broiler chicks were purchased from a commercial hatchery. Before the experiment, birds were subjected to a 3-day adaptation period to achieve a mean body weight (BW) of 92.68 ± 0.17 g. Then the birds were randomly allotted into 4 treatments (10 replications/TRT, 10 chicks/replicate). The birds were fed baseline diets supplemented with MUR at four levels: 0, 200, 400, and 600 mg Kg^−1^ diet, with enzyme activity 0, 12,000, 24,000, and 36,000 LSU(F)/kg diet, respectively. Feed and water were provided *ad libitum* to the birds. The proximate chemical composition of the basal diet during the starter (4–10 days), grower (11–23 days), and finisher periods (24–35 days) is shown in Table 1. Administrative conditions and experimental diets were carried out according to the Ross 308 broiler feeding specification AVIAGEN [27]. Chicks were raised in an open, well-ventilated building with sawdust (7 birds/m^2^) where the temperature was pointed at 34 °C during the 1 week and regularly lowered till it reached 25 °C at the end of the experiment. Standard vaccination programs were implemented against Newcastle (on days 4 and 14) and Gumboro diseases (on days 7 and 22).

### 2.2. Growth Performance

Chicks were weighed at the beginning of the experiment (day 4) to determine the initial body weight (IBW). Then, the body weight and feed intake (FI) were measured at the end of each feeding period to calculate BW gain (BWG), FI, and feed conversion ratio (FCR).

$$FCR=\frac{FI\;(g)}{BWG\;(g)}$$

The protein efficiency ratio (PER) was calculated according to McDonald et al. [28].

$$PER=\frac{Live\;weight\;gain\;(g)}{Protein\;intake\;(g)}$$

The relative growth rate (RGR) was calculated according to Brody [29].

$$RGR=\frac{FinalBW-IBW}{0.5\left(IBW+FinalBW\right)}\times 100$$

### 2.3. Carcass Measurements

Ten chicks in each group were euthanized using cervical dislocation at the end of this study [30]. A trained descriptive panel of 5 persons identified the sensory profiles (color, odor, and consistency) of the muscles examined as given a score from 1 to 5, where 5 represents normal, 4 represents a minor deviation, 3 represents a moderate deviation, 2 represents a high deviation, and 1 represents extremely deviated. The hot carcass weight was recorded. The cut and gutted carcasses were deprived of their heads, feathers, feet, internal organs such as the liver, spleen, heart, gizzard, digestive system, and abdominal fat before being weighed. The carcass weight was measured, and the carcass yield percentage was estimated as follows:

$$Carcass\;yield\%=\frac{Carcass\;weight,g}{Live\;BW,g}\times 100$$

#### 2.3.1. Color Evaluation

A reflectance colorimeter 2 was used to quantify the lightness, redness, and yellowness components of the CIE system color profile while using illumination source C [31]. Throughout the investigation, the colorimeter was calibrated using a typical white ceramic floor. A region free of evident color defects (bruises, discolorations, hemorrhages, full blood vessels, picking damage, or any other condition that may have impacted a uniform color reading) was selected for color measurement on the cranial, medial surface (bone side).

#### 2.3.2. Texture Evaluation

Using a Warner Bratzler (WB) single-blade attachment on a Model 1122 Instron Universal testing equipment, the shear value of the breast muscle was calculated according to Lyon and Lyon [32].

### 2.4. Sampling

We randomly selected ten birds/group and euthanized them by cervical dislocation at the end of the experiment [30]. Blood was collected without anticoagulant and centrifuged at 3500 rpm for 15 min. The separated serum was kept at −20 °C till biochemical analysis. Breast muscles were sampled for fatty acid analysis. Spleen samples were collected for immunohistochemistry. Samples from different sections of the small intestine (duodenum, jejunum, and ileum) were taken for histological evaluation.

### 2.5. Fatty Acid Profile of Breast Muscles

We extracted the oils from the breast muscle (5 samples/group) with a solvent mixture of chloroform/methanol (2:1, *v*/*v*) [33]. We measured the fatty acids profile in the extracted oil according to AOAC [34].

### 2.6. Intestinal Histology

Small intestine samples (*n* = 10, 2 cm) were preserved in 10% neutral buffered formaldehyde for 72 h, dehydrated, cleared, embedded in wax, sliced with a microtome (Leica RM 2155, Wetzlar, Germany) into 4 µm cross-sections and longitudinal sections, and stained using hematoxylin and eosin (H&E) [35]. The morphometric measures were determined as explained by Amer et al. [36]

### 2.7. Serum Lipid Profile

We used Bio-spectrum colorimetric diagnostic kits (Egyptian Company for Biotechnology, Cairo, Egypt) the determine the serum levels of total cholesterol (TC), triglycerides (TG), and high-density lipoprotein cholesterol (HDL-C). Low-density lipoprotein-cholesterol (LDL-C) was calculated by the Iranian formula, LDL-C= TC/1.19 + TG/1.9–HDL/1.1–38. Using the turbidimetry method [37], the very low-density lipoprotein cholesterol (VLDL-C) was estimated.

### 2.8. Immune Indices and Protein Gram

We measured the serum level of complement 3 (C3) using a sandwich enzyme-linked immunosorbent assay (ELISA kit, CAT. NO. LS-F9287 Life Span Biosciences, Inc., Seattle, WA, USA). The serum lysozyme activity was determined according to Lie et al. [38]. We measured the serum interleukin 10 (IL10) level using chicken ELISA kits (CAT.NO. MBS701683, MyBioSource Co., San Diego, CA, USA).

According to Grant [39] and Doumas et al. [40], we measured the serum total protein and albumin levels, respectively. The globulin serum level was mathematically determined by subtracting albumin values from the total proteins [41].

### 2.9. Immunohistochemistry

Ten spleen samples/group were used to assess the immunoexpression of CD3 and CD20, according to Saber et al. [42]. Slides were treated with mouse anti-Chicken CD3, clone CT-3 (Bio-Rad Lab., Dubai, United Arab Emirates), and CD20 (ThermoFisher Scientific, Waltham, MA, USA) and examined as described by Amer et al. [36]. The median grayscale is used to express immunoreactive intensity [43].

### 2.10. Statistical Analysis

The statistical analysis was performed using a completely randomized design and the general linear model (GLM) procedure of SAS 9.2. The linear and quadratic effects of increasing inclusion levels were determined using orthogonal polynomial contrasts. Data variance was expressed as pooled SEM, and the significance level was set at *p* < 0.05.

Statistical model:

Yik = U + Ti + Eijk

where Yik = observed value of the response variable, U = observed mean for the response variable, Ti = the fixed effect of the treatment group, and Eijk = random error.

## 3. Results

### 3.1. Growth Performance

Adding MUR to broiler meals in amounts of 200, 400, or 600 mg/kg had no impact on BW, BWG, and FI (*p* > 0.05) during the periods from 4–10, 11–23, and 24–35 days of age. MUR quadratically impacted the FCR of broiler chicks at 11 and 23 days of age (*p* = 0.02; Table 2).

### 3.2. Carcass Traits

**Figure 1:**
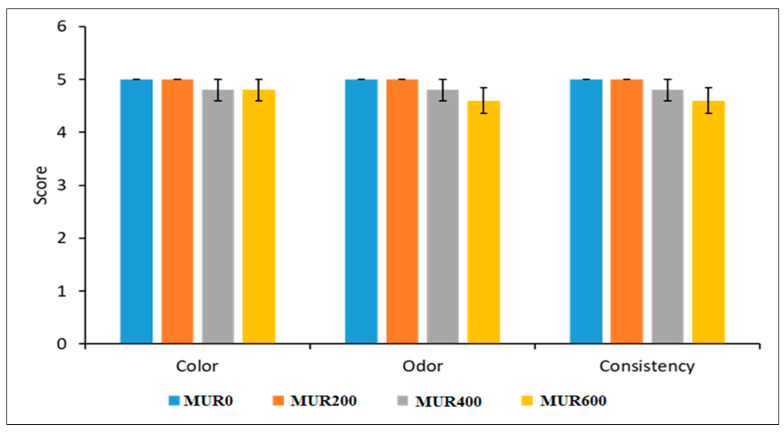
The effect of MUR on the sensory characteristics of the broiler carcasses. Values represent mean ± SE of the scores of the five panelists. Bars without superscripts are not significantly different (Tukey’s test, *p* > 0.05).

Sensory characteristics, including color, odor, shear value for texture evaluation, and consistency, did not show any significant variations (*p* > 0.05) among the treatments (Figure 1, Table 3). The weights of the internal organs (gizzard, intestine, bursa, spleen, carcass yield, and liver) were not significantly different between the experimental groups (*p* > 0.05; Table 4).

### 3.3. Fatty Acid Profile of the Breast Muscle

The percentages of α-linolenic acid, docosahexaenoic acid, arachidonic acid, n−3 PUFA, and n−6 PUFA were increased linearly in the breast muscles of birds fed MUR-supplemented diets (*p* ≤ 0.01). The percentages of eicosapentaenoic acid, docosapentaenoic acid, and linoleic acid were increased (Lin. and Quad. *p* < 0.05) in the breast muscles of birds fed MUR-supplemented diets (Table 5).

### 3.4. Intestinal Histology and Morphometric Measures

**Figure 2:**
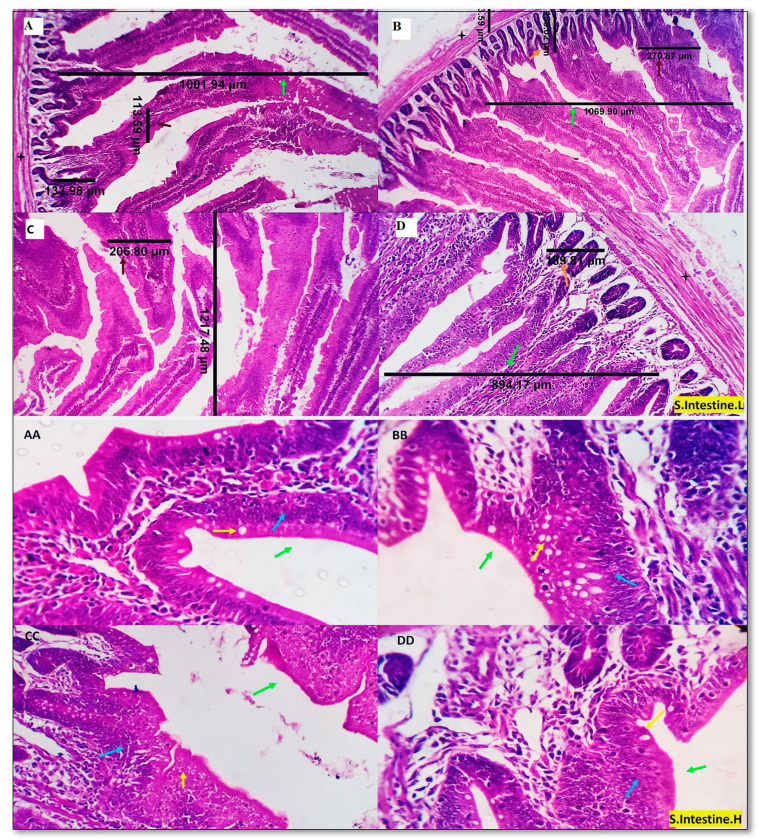
Photomicrographs from the small intestine (duodenum) of the experimental treatments display the different morphometric measures; VH (green arrows), VW (brown arrows), CD (orange arrows), and MCT (black stars). A moderate number of goblet cells (yellow arrows) in groups AA and BB (27 and 29 cells/HPF, respectively) and comparatively higher numbers in groups CC and DD (39 and 31 cells/HPF, respectively). Mild villous epithelial stratification with multilayered proliferate cells, central rounded nuclei, and abundant eosinophilic cytoplasm (blue arrows) can be seen in (groups BB and CC). The standard arrangement of the villous cells is observable in other groups. H&E X 100, 200. MUR0: (**A**,**AA**), MUR200: (**B**,**BB**), MUR400: (**C**,**CC**), MUR600: (**D**,**DD**) low and high power, respectively.

The intestinal histology of the control and the experimental treatments is displayed in Figure 2. Normal intestinal histomorphology was observed in the control group. The intestinal morphology of the experimental treatments (MUR200, MUR400, and MUR600 groups) also appeared normal. In addition, mild villous epithelial stratification with multilayered proliferating cells, showing central rounded nuclei and abundant eosinophilic cytoplasm, was seen in the MUR200 and MUR400 groups. The typical arrangement of the villous epithelial cells could be observed in the other groups.

The dimensions of the villous width (VW), villous height (VH), the crypt of Lieberkühn depth (CD), and muscular coat thickness (MCT) were recorded in the duodenum, jejunum, and ileum with the best dimensions were seen in MUR200 and MUR400 groups followed by MUR0 and MUR600 groups (Table 6). In detail, the duodenal VH and ileal CD were increased in the MUR2 and MUR400 groups (*p* < 0.01). The jejunal VH was higher in the MUR400 group (*p* < 0.01). The duodenal VW, jejunal VW, and ileal VH and VW were increased in all MUR-supplemented groups (*p* < 0.01). The duodenal CD and MCT were raised in the MUR200 and MUR 600 groups (*p* < 0.01). The VH:CD ratio was higher in the MUR400 group in the duodenum, the MUR200 and MUR400 groups in the jejunum, and the MUR400 and MUR600 in the ileum (*p* < 0.01). The jejunal CD and MCT were decreased by MUR supplementation (*p* < 0.01). The ileal MCT was increased in the MUR600 group and lowered in the MUR200 group (*p* < 0.01). Moderate goblet cell count in the duodenum was reported in MUR0 and MUR200 groups (27 and 29 cells/HPF, respectively) and comparatively higher numbers in MUR400 and MUR600 groups (39, 31 cells/HPF, respectively).

### 3.5. Serum Lipid Profile

Adding MUR at 200, 400, and 600 mg kg^−1^ linearly reduced the TC, TG, and LDL-C levels. Still, they significantly increased the HDL-C and VLDL-C compared with the MUR0 group (linear *p* < 0.01; Table 7).

### 3.6. Immune Indices

Compared to controls, there was a substantial rise in the blood concentration of total protein, albumin, globulin, and lysozyme activity as MUR levels increased (linear *p* < 0.01). In addition, linear and quadratic increase in the IL10 and complement 3 levels in the serum of birds fed MUR-supplemented diets (*p* < 0.05; Table 8).

### 3.7. Immunohistochemical Analysis and Morphometric Measures

**Figure 3:**
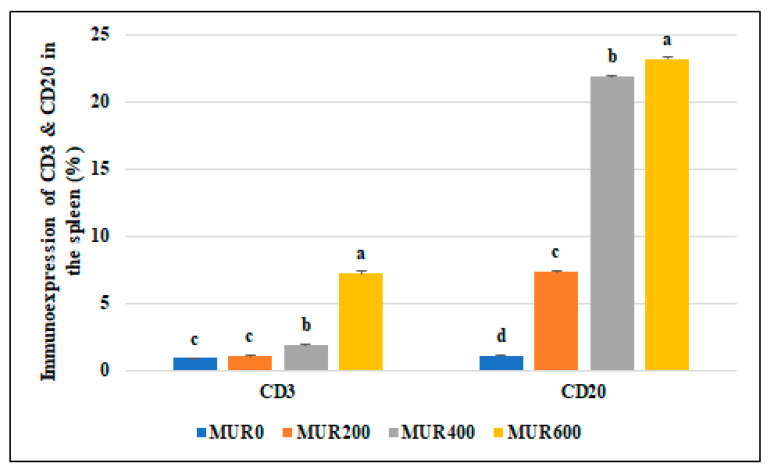
The morphometric analytic data of immunostained CD3 and CD20 cells in the spleen of different experimental groups. Means with different superscripts (^a, b, c, d^) are significantly different at *p* < 0.05.

**Figure 4:**
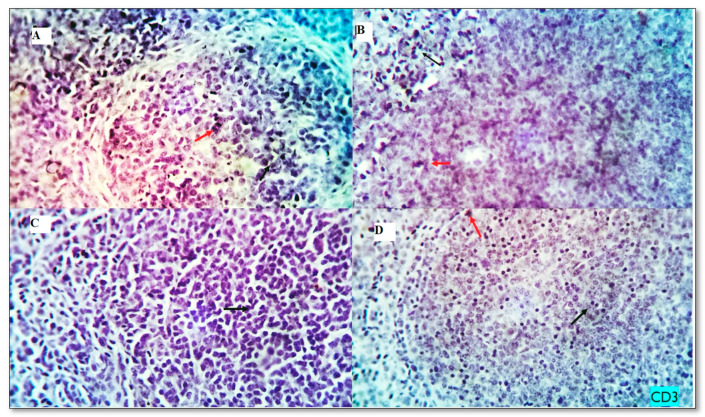
The immunostained positive CD3 cells (red arrows) and negative cells (black arrows) in the spleen of different experimental groups. (**A**): MUR0, (**B**): MUR200, (**C**) MUR400 and (**D**): MUR600.

**Figure 5:**
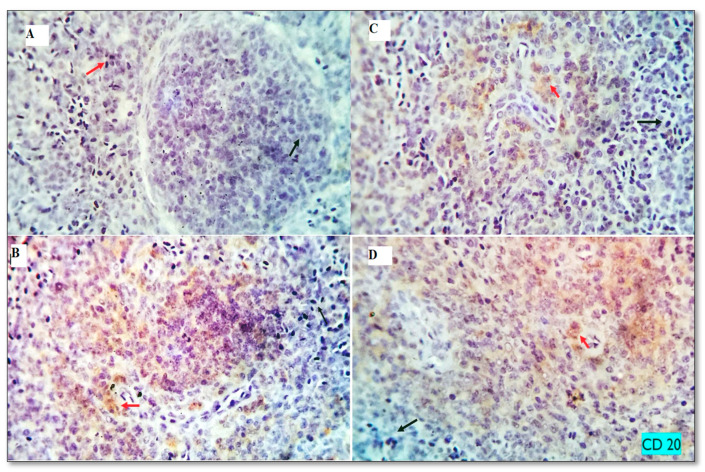
The immunostained positive CD20 cells (red arrows) and negative cells (black arrows) in the spleen of different experimental groups. (**A**): MUR0, (**B**): MUR200, (**C**) MUR400 and (**D**): MUR600.

The baseline immune cell populations in the spleen of broiler chickens fed different levels of MUR (0, 200, 400,600 mg kg^−1^) were examined using leukocyte-specific markers (CD3 and CD20). Examined spleen sections showed an average percentage of positive cells per 3 high power fields (HPF) to CD3 (T- cell marker) as follows; 0.93, 1.13 1.86, and 7.22% for MUR0, MUR200, MUR400, MUR600 groups, respectively (Figure 3 and Figure 4). An average percentage of positive cells per 3 HPF to CD20 (B—cell marker) was 1.13, 7.39, 21.88, and 23.20% for MUR0, MUR200, MUR400, MUR600 groups, respectively (Figure 3 and Figure 5).

## 4. Discussion

In the current investigation, adding MUR to broiler chickens' diets had no significant effect on their growth. A significant decrease in FCR was noted when MUR was added at various levels [9,18]. Other studies revealed similar impacts on growth performance when other muramidase from multiple origins were added to broiler chicken diets. Chickens fed a standard meal that contained 10% transgenic rice expressing lysozyme had better feed efficiency [44]. Gong et al. [10] observed that adding lysozyme to broiler diets did not influence growth performance. Goes et al. [25] reported improved FCR in broiler chickens fed a diet included with MUR throughout the whole experimental period and improved BWG and FCR of intestinally challenged chickens fed on MUR (35,000 LSU (F)/kg). They also established that MUR supplementation reduced intestinal permeability, enhanced intestinal barrier function, increased nutrient digestibility, and raised the total carotenoids in blood. They attributed the improved nutrient digestibility by MUR to the improved intestinal health and function and its synergism with other feed enzymes. In the study of Brugaletta et al. [45], they evaluated two concentrations of MUR; low-dose 25,000 or high-dose 45,000 LSU(F)/kg feed in diets of broiler chickens. They showed that the high-dose group resulted in higher feed intake and body weight and improved FCR than the control group, while the low-dose group showed intermediate performance. The inability of MUR to increase birds’ weight in the present study compared to the previously-mentioned studies may be due to the redirection of available nutrients to the immune enhancement and modifications in the intestinal morphology (reported in the current study) rather than improving the bird’s growth. The current study also demonstrated MUR’s nonsignificant effect on carcass traits and their sensory characteristics. Taylor et al. [46] reported that muramidase is an ideal alternative to conventional antimicrobials for organic meat and poultry production, with no deviations in the sensory characteristics of the final products.

Several studies want to distinguish between herbs influencing fatty acid properties and antioxidant status [36,47,48]. One of the critical elements affecting consumer health is the quality of poultry meat [49,50]. Because breast meat includes more PUFA and less saturated FA than other varieties of animal meat, such as beef and lamb, it can be considered a crucial element of a balanced diet [51]. Additionally, its consumption is rising globally due to how well it complements contemporary cooking techniques. In the current study, the percentages of n−3 and n−6 PUFAs in breast muscle were considerably and level-dependently increased by MUR added to the diets.

In the current study, there was a significant rise in the blood levels of TC, TG, and LDL-C as MUR levels increased while considerably raising HDL and VLDL-C. Information about the effect of MUR on blood lipid profile is not well documented, so we referred to other enzymes in our study. Goli and Aghdam Shahryar [52] showed a reduction in the TC, LDL-C, and TG and a rise in the HDL-C by adding multienzymes (xylanase, β-glucanase, pectinase, and cellulase) at different stages of growth of broiler chickens.

The basis of optimal poultry performance is a healthy and functioning gut. If gut health and function are diminished, nutrient digestion and absorption are altered, and growth performance may be affected. Efficient gastrointestinal (GI) function and health are significant factors in attaining animal welfare, feed efficiency, and sustainability [2]. The current study recorded the VW, VH, CD, and MCT dimensions in the duodenum, jejunum, and ileum. The best dimensions were seen in MUR200 and MUR400 groups, respectively, followed by MUR0 and MUR600 groups. The VH: CD ratio in the duodenum was increased in MUR400 and decreased in the MUR200 and MUR600 groups, while in the jejunum, it was increased in MUR200–400 and decreased in MUR600. In the ileum, it was increased with increasing the level of MUR. This could reflect the intestinal response to MUR dietary addition. These responses to the different MUR levels may be due to the changes in the intestinal microbiota, higher PGNs from an increased bacterial turnover, or other metabolites [5]. In the experiment of Boroojeni et al. [18], the morphometric variables and the lymphocytes (CD45) or goblet cell numbers in the jejunum are not affected by MUR. However, MUR addition raised VH: CD ratio and reduced the CD45 cell number in the ileum. Sais et al. [5] showed no significant effect of MUR supplemented at 35,000 LSU (F)/kg on the VH, CD, nor VH: CD ratio in the jejunum of broiler chickens. In the study of Humphrey et al. [44], birds fed a diet including 10% modified rice expressing lysozyme revealed increased duodenal VH and decreased leukocyte number in the ileal lamina propria. Adding HEW lysozyme to broiler diets raised the VH and CD in the jejunum [15]. HEW lysozyme supplementation (90 mg/kg) in weaning piglet diets increased VH: CD ratio in the duodenum and jejunum with no effect on VH and CD in the duodenum, jejunum, and ileum [53]. Liquid HEW lysozyme (100 mg/L) supplementation to milk replacer in weaning piglets increased CD in the jejunum, ileum, and VH in the ileum [12]. Weaned pigs fed diets including a water-soluble HEW lysozyme exhibited taller villi in the ileum [54]. The improved membrane integrity, nutrient digestion, and absorption by MUR are likely due to its effect on bacterial PGN hydrolysis [25].

Bacterial cell walls (PGNs) have rigid structures that are hard to break and are created by continual N-acetylmuramic acid sequences linked with N-acetyl glucosamine by β-1,4 bonds [55]. In a typical conventional gastrointestinal environment, where commensals are predominant, PGNs can be amassed, and a PGN-rich state can be established [56]. Though, microbial cellular debris accumulation can lead to the formation of a layer on the intestinal epithelium, which impairs appropriate nutrient absorption [18,56]. PGN can be identified as a microbe-associated molecular pattern (MAMP) by immune cells that can initiate inflammation when identified by toll-like receptors 2 appearing in the cell membrane of immune cells [57]. As a result, increased expression of pro-inflammatory mediators, antimicrobial peptides, anti-apoptotic factors, and a defensive reaction to the infecting microbe [58].

The PGN polymer is degraded during the growth and development by various PGN hydrolases, such as peptidases, muramidase, amidase, and glucosamidase (produced by the host and the associated microbiota). Consequently, muropeptides (hydrolyzed products of bacterial PGNs) are eliminated from the cell wall into the gut [7]. It has been demonstrated that muropeptides alter inflammatory responses in the gastrointestinal tract [8]. Therefore, it can be hypothesized that the PGN hydrolysis by dietary MUR and muropeptide production could probably modulate the inflammatory response in the gastrointestinal tract, explaining the advantages reflected on gastrointestinal health and function. So, lysis of PGN by MUR has the benefits of reducing inflammation, improving nutrient absorption, and redirecting nutrients to animal growth. Murimyl dipeptide, a hydrolysis product, is a strong adjuvant efficient in increasing immunoglobulin A (IgA) secretion and fast bacterial clearance in vivo [59]. Lysozyme can bind to the lipid A part of bacterial endotoxin [60], which leads to a conformational shift that prevents endotoxin from interrelating with macrophage receptors and inhibits the production of the pro-inflammatory cytokines such as interleukin-1 (IL-1), interleukin-6 (IL-6), and tumor necrosis factor-alpha (TNF-α) [61,62].

The present study showed a substantial and level-dependent rise in the blood concentration of total protein, albumin, globulin, IL10, and complement 3 levels and lysozyme activity in the serum of birds fed diets supplemented with MUR. Moreover, MUR dietary addition significantly increased the immunoexpression of C3 and CD20 in the spleen tissues. Lymphocyte subpopulations are identified by the expression of specific cell surface biomarkers. After antigen recognition, helper and cytotoxic T cells are activated through a signaling cascade initiated by the T-cell co-receptor CD3 [63]. Regulation of the B-cell activity, differentiation, and proliferation is the function of B-lymphocyte surface antigen CD20 [64]. The present study indicates the immunomodulatory effects of MUR addition. In broilers challenged with *Eimeria maxima* and *C. perfringens,* adverse health effects from necrotic enteritis were reduced by feeding on diets containing lysozyme-based antimicrobial blends [65]. MUR was also reported to enhance the antimicrobial activity of lactoferrin and specific antibodies [66].

## 5. Conclusions

The current study evaluated the dietary addition of MUR (0, 200, 400, 600 mg Kg^−1^, with enzyme activity 0, 12,000, 24,000, and 36,000 LSU(F)/kg diet, respectively) in broiler chicken diets. The results concluded that MUR addition resulted in significant intestinal responses in terms of VW, VH, CD, and MCT dimensions in the duodenum, jejunum, and ileum, with the best dimensions seen in the MUR200 and MUR400 groups, without affecting the bird’s growth. MUR-supplemented diets helped enrich the breast meat of broiler chickens with n−3 and n−6 PUFA without altering the sensory characteristics of the carcass, which could increase consumer acceptance. Moreover, Increased supplementation levels of MUR reduced the total cholesterol, triglycerides, and LDL-C while considerably raising HDL and VLDL cholesterol, indicating a hypolipidemic effect. Dietary MUR boosted the birds’ immune systems by improving the blood proteinogram, increasing lysozyme activity, IL10 and C3 concentrations, and upregulating CD3 and CD20 immunoexpression in the spleen tissues.

## Figures and Tables

**Table 1:** The proximate chemical composition of the basal diet as fed basis (g Kg ^−1^).

Ingredients	Starter Period	Grower Period	Finisher Period
Yellow corn	559	593	62.27
Soybean meal, 48%	337	281	236
Corn gluten, 60%	37.3	53	60
Soybean oil	22	30	40
Calcium carbonate	12	12	11
Calcium dibasic phosphate	15	14	13
Common salt	1.5	1.5	1.5
Premix *	3	3	3
DL-methionine, 98%	4	3	3.3
Lysine HCl, 78%	4.7	4.5	4
Choline	0.7	0.7	0.7
Threonine	1	1	1
Phytase	0.05	0.05	0.05
Na_2_Co_3_	2.5	2.5	2.5
Antimycotoxin	1	1	1
Chemical composition (g Kg^−1^)			
ME (Kcal/kg)	3003	3101	3202
Crude protein	231	215.3	200.4
Calcium	9.41	9.04	8.32
Available phosphorus	4.82	4.49	4.17
Lysine	14.7	13.1	11.5
Digestible lysine	13.40	12.11	10.66
Methionine	7.20	6.10	6.00
Digestible methionine	6.90	5.80	5.73
Threonine	9.90	9.12	7.84
Digestible threonine	8.70	8.02	6.93

* Premix per kilogram of diet: 10 mg pantothenic acid; 1.8 mg thiamine; 3.6 mg riboflavin; 35 mg niacin; 0.15 mg biotin; 0.01 mg cobalamin; 0.55 mg folic acid; 3.5 mg pyridoxine; 1500 IU Vit. A; 200 IU Vit. D; 10 mg Vit. E; 0.5 mg vitamin K; 8 mg copper; 40 mg zinc; 80 mg iron; 60 mg manganese; 40 mg; Se, 0.15 mg I, 0.35 mg.

**Table 2:** The effect of MUR on the broiler chickens’ growth.

Parameters	MUR (mg Kg^−1^)				SEM	Regression	
	0	200	400	600		Linear	Quadratic
IBW (g)	93	92.08	93.12	92.5	0.17	0.72	0.61
Starter period							
BW(g)	334	338	328	334	2.30	0.63	0.73
BWG(g)	242	245	235	242	2.36	0.65	0.76
FI (g)	264	260	259	260	1.71	0.41	0.50
FCR	1.09	1.06	1.10	1.07	0.01	0.86	0.85
Grower period							
BW(g)	1168	1155	1151	1171	12.40	0.97	0.57
BWG(g)	834	817	823	837	11.80	0.90	0.59
FI (g)	1137	1145	1199	1104	17.68	0.78	0.16
FCR	1.36	1.40	1.45	1.32	0.02	0.56	0.02
Finisher period							
BW(g)	2020	2005	1977	2006	24.17	0.78	0.71
BWG(g)	851	850	826	834	22.97	0.76	0.93
FI(g)	1507	1503	1555	1417	27.97	0.40	0.26
FCR	1.79	1.77	1.88	1.72	0.06	0.83	0.59
Overall performance							
BW(g)	2020	2005	1977	2006	24.17	0.78	0.71
BWG(g)	1927	1913	1884	1913	24.19	0.78	0.71
FI (g)	2909	2908	3014	2782	45.49	0.51	0.23
FCR	1.51	1.52	1.60	1.45	0.02	0.65	0.13
PER	3.16	3.15	3.00	3.29	0.05	0.61	0.14
RGR	182.3	182.4	182	182.3	0.20	0.81	0.80

Means without superscripts are not significantly different (Tukey’s test, *p* > 0.05). IBW: initial body weight, BW: body weight, BWG: body weight gain, FI: feed intake, FCR: feed conversion ratio, PER: protein efficiency ratio, RGR: relative growth rate.

**Table 3:** Effect of MUR on the color and texture of breast muscles.

Traits	MUR (mg Kg^−1^)				SEM	Regression	
	0	200	400	600		Linear	Quadratic
Lightness	52.00	53.00	51.00	54.00	2.17	0.65	0.68
Redness	2.04	2.02	1.99	1.95	0.20	0.45	0.35
Yellowness	1.55	1.56	1.52	1.49	0.30	0.52	0.15
Shear value (Kg)	2.44	2.35	2.18	2.45	0.36	0.70	0.08

Means without superscripts are not significantly different (Tukey’s test, *p* > 0.05).

**Table 4:** The effect of MUR on the carcass characteristics relative to the live Wt. (%).

Items	MUR (mg Kg^−1^)				SEM	*p*-Value	
	0	200	400	600		Lin.	Quad.
Carcass yield %	59.58	59.39	60.90	61.52	0.72	0.32	0.80
Gizzard	2.05	1.84	2.10	1.66	0.12	0.47	0.67
Intestine	5.57	5.33	5.06	5.19	0.19	0.47	0.67
Liver	2.54	2.10	2.17	2.22	0.11	0.39	0.31
Bursa	0.17	0.13	0.13	0.16	0.01	0.49	0.06
Spleen	0.12	0.08	0.08	0.12	0.01	0.78	0.05

Means without superscripts are not significantly different (Tukey’s test, *p* > 0.05).

**Table 5:** The effect of MUR on the breast muscle fatty acid profile (% of total fatty acids).

Items	MUR (mg Kg^−1^)				SEM	*p*-Value	
	0	200	400	600		Lin.	Quad.
18:3 n−3	0.032 ^b^	0.055 ^a^	0.065 ^a^	0.077 ^a^	0.005	<0.01	0.07
20:5 n−3	0.021 ^b^	0.053 ^a^	0.056 ^a^	0.066 ^a^	0.004	<0.01	0.04
22:5 n−3	0.022 ^b^	0.043 ^a^	0.051 ^a^	0.053 ^a^	0.004	<0.01	0.02
22:6 n−3	0.023 ^b^	0.044 ^a^	0.035 ^ab^	0.048 ^a^	0.003	0.01	0.14
18:2 n−6	0.790 ^b^	0.851 ^ab^	0.901 ^a^	0.872 ^a^	0.01	<0.01	0.02
20:4 n−6	1.18 ^b^	1.27 ^ab^	1.26 ^ab^	1.31 ^a^	0.02	0.01	0.39
n−3 (%)	0.104 ^b^	0.205 ^a^	0.192 ^a^	0.235 ^a^	0.016	<0.01	0.08
n−6 (%)	1.97 ^b^	2.12 ^a^	2.16 ^a^	2.18 ^a^	0.025	<0.01	0.03

18:3 n−3: α-linolenic acid, 20:5 n−3: eicosapentaenoic acid, 22:5 n−3: docosapentaenoic acid, 22:6 n−3: docosahexaenoic acid, 18:2 n−6: Linoleic acid, 20:4 n−6: arachidonic acid, n−3: omega 3 PUFA, n−6: omega 6 PUFA. Means with different superscripts (^a, b^) are significantly different (Tukey’s test, *p* < 0.05).

**Table 6:** The effect of MUR on the intestinal morphometric dimensions.

	MUR (mg Kg^−1^)				SEM	Regression	
	0	200	400	600		Linear	Quadratic
Duodenum							
VH (µm)	985 ^c^	1052 ^b^	1197 ^a^	838 ^d^	33.5	<0.01	<0.01
VW (µm)	106 ^d^	265 ^a^	202 ^b^	188 ^c^	14.7	<0.01	<0.01
CD (µm)	125 ^c^	163 ^b^	109 ^d^	186 ^a^	7.94	<0.01	<0.01
MCT (µm)	70.7 ^c^	109 ^b^	83.6 ^bc^	299 ^a^	24.0	<0.01	<0.01
VH: CD	7.91 ^b^	6.44 ^c^	10.90 ^a^	4.51 ^d^	0.609	<0.01	<0.01
Jejunum							
VH (µm)	997 ^b^	907 ^c^	1059 ^a^	701 ^d^	35.1	<0.01	<0.01
VW (µm)	94.1 ^d^	131 ^c^	181 ^a^	164 ^b^	8.65	<0.01	<0.01
CD (µm)	176 ^a^	99.2 ^d^	128 ^c^	162 ^b^	7.81	0.02	<0.01
MCT (µm)	156 ^a^	68.6 ^c^	93.7 ^b^	98.1 ^b^	8.29	<0.01	<0.01
VH: CD	5.63 ^c^	9.14 ^a^	8.26 ^b^	4.32 ^d^	0.502	<0.01	<0.01
Ileum							
VH (µm)	442 ^c^	559 ^b^	839 ^a^	571 ^b^	37.5	<0.01	<0.01
VW (µm)	121 ^b^	144 ^a^	112 ^b^	98.3 ^c^	4.46	<0.01	<0.01
CD (µm)	113 ^c^	172 ^b^	194 ^a^	112 ^c^	9.38	0.11	<0.01
MCT (µm)	123 ^b^	107 ^c^	117 ^b^	175 ^a^	6.86	<0.01	<0.01
VH: CD	3.90 ^c^	3.25 ^d^	4.32 ^b^	5.07 ^a^	0.176	<0.01	<0.01

Means with different superscripts (^a, b, c, d^) are significantly different (Tukey’s test, *p* < 0.05). VW: villous width, VH: villous height, CD: the crypt of Lieberkühn depth, and MCT: muscular coat thickness. VH:CD: villous height: crypt depth ratio.

**Table 7:** Effect of MUR on serum lipid profile of broiler chickens.

Items	MUR (mg Kg^−1^)				SEM	*p*-Value	
	0	200	400	600		Lin.	Quad.
TC (mmol/L)	3.56 ^a^	3.44 ^b^	3.43 ^b^	3.40 ^b^	0.024	<0.01	0.079
HDL (mmol/L)	2.00 ^b^	2.12 ^ab^	2.19 ^a^	2.53 ^a^	0.040	<0.01	0.451
LDL (mmol/L)	1.36 ^a^	1.09 ^b^	1.00 ^b^	0.90 ^b^	0.043	<0.01	0.073
VLDL (mmol/L)	0.193 ^b^	0.223 ^ab^	0.230 ^ab^	0.247 ^a^	0.009	<0.01	0.506
TG (mmol/L)	1.34 ^a^	1.23 ^b^	1.20 ^bc^	1.16 ^c^	0.011	<0.01	0.018

Means with different superscripts (^a, b, c^) are significantly different (Tukey’s test, *p* < 0.05). TC: total cholesterol, TG: triglycerides, HDL-C: high-density lipoprotein cholesterol, LDL-C: low-density lipoprotein-cholesterol.

**Table 8:** Effect of MUR on serum immune indices of broiler chickens.

Items	MUR (mg Kg^−1^)				SEM	*p*-Value	
	0	200	400	600		Lin.	Quad.
Total protein (g/dL)	3.11 ^c^	3.43 ^bc^	4.19 ^b^	5.34 ^a^	0.19	<0.01	0.072
Albumin (g/dL)	1.19 ^b^	1.30 ^b^	1.52 ^b^	2.13 ^a^	0.12	<0.01	0.091
Globulin (g/dL)	1.92 ^c^	2.12 ^bc^	2.67 ^b^	3.21 ^a^	0.013	<0.01	0.243
Lysozyme (µg/mL)	129 ^c^	161 ^b^	185 ^ab^	193 ^a^	6.33	<0.01	0.094
IL10 (pg/mL)	1.80 ^b^	3.70 ^a^	3.71 ^a^	3.90 ^a^	4.43	<0.01	0.039
Complement 3 (mg/dL)	1.07 ^c^	1.22 ^b^	1.28 ^ab^	1.34 ^a^	0.015	<0.01	0.014

SEM: standard error of means. Lin and Quad: linear and quadratic responses, respectively, to supplementation levels of microbial muramidase. Means with different superscripts (^a, b, c^) are significantly different (Tukey’s test, *p* < 0.05).

## Data Availability

The datasets generated or analyzed during the current study are not publicly available but are available from the corresponding author upon reasonable request.
